# A cross-sectional analysis of syncytiotrophoblast membrane extracellular vesicles–derived transcriptomic biomarkers in early-onset preeclampsia

**DOI:** 10.3389/fcvm.2023.1291642

**Published:** 2023-11-30

**Authors:** Toluwalase Awoyemi, Wei Zhang, Maryam Rahbar, Adam Cribbs, Prasanna Logenthiran, Shuhan Jiang, Gavin Collett, Ana Sofia Cerdeira, Manu Vatish

**Affiliations:** ^1^ Nuffield Department of Women’s & Reproductive Health, University of Oxford, Oxford, United Kingdom; ^2^ Nuffield Department of Orthopaedics, Rheumatology and Musculoskeletal Sciences, University of Oxford, Oxford, United Kingdom

**Keywords:** transcriptomics, syncytiotrophoblast membrane extracellular vesicles (STB-EVs), preeclampsia, biomarkers, placenta EVs, mechanisms

## Abstract

**Background:**

Preeclampsia (PE) is a pregnancy-specific hypertensive disorder affecting 2%–8% of pregnancies worldwide. Biomarker(s) for the disorder exists, but while these have excellent negative predictive value, their positive predictive value is poor. Extracellular vesicles released by the placenta into the maternal circulation, syncytiotrophoblast membrane extracellular vesicles (STB-EVs), have been identified as being involved in PE with the potential to act as liquid biopsies.

**Objective:**

The objective of this study was to identify the difference in the transcriptome of placenta and STB-EVs between preeclampsia and normal pregnancy (NP) and mechanistic pathways.

**Methods/study design:**

We performed RNA-sequencing on placental tissue, medium/large and small STB-EVs from PE (*n* = 6) and NP (*n* = 6), followed by bioinformatic analysis to identify targets that could be used in the future for EV-based diagnostic tests for preeclampsia. Some of the identified biomarkers were validated with real-time polymerase chain reactions.

**Results:**

Our analysis identified a difference in the transcriptomic STB-EV cargo between PE and NP. We then identified and verified the differential expression of *FLNB*, *COL17A1*, *SLC45A4*, *LEP*, *HTRA4*, *PAPP-A2*, *EBI3*, *HSD17B1*, *FSTL3*, *INHBA*, *SIGLEC6*, and *CGB3*. Our analysis also identified interesting mechanistic processes via an *in silico* prediction of STB-EV-based mechanistic pathways.

**Conclusions:**

In this study, using comprehensive profiling of differentially expressed/carried genes of three linked sample subtypes in PE, we identified potential biomarkers and mechanistic gene pathways that may be important in the pathophysiology of PE and could be further explored in future studies.

## Introduction

Preeclampsia (PE) is a multisystemic hypertensive disorder that affects approximately 2%–8% of pregnancies worldwide (1). It is a pregnancy-specific complication that results in hypertension (systolic blood pressure ≥ 140 mmHg/diastolic pressure ≥ 90 mmHg) and proteinuria (protein/creatinine ratio of ≥ 30 mg/mmol or more), or evidence of maternal acute kidney injury, liver dysfunction, neurological abnormalities, hemolysis, thrombocytopenia, and/or fetal growth restriction, and, in severe cases, death (2). Preeclampsia, in particular, early-onset preeclampsia (EOPE), has been referred to as a two-stage process, starting with malplacentation that results in syncytiotrophoblast stress (stage 1) and ending in maternal end-organ damage (stage 2) (3). However, the direct cause of PE (i.e., mechanisms of placental dysfunction) is still under study, with the only available treatment being the delivery of the placenta (4).

Syncytiotrophoblast membrane extracellular vesicles (STB-EVs) are lipid bilayer spherical structures of placental origin that can be classified based on their size and biogenesis pathways into medium/large and small STB-EVs (5). Small STB-EVs are less than or equal to 200 nm in size and are formed through multivesicular bodies, whereas medium/large EVs are between 201 and 1,000 nm in size and are released directly through the budding of the plasma membrane (6). Their cargo consists of surface proteins, encapsulated proteins, and different subclasses of RNAs such as long non-coding RNAs, messenger RNAs, transfer RNAs, and micro-RNAs (7), which may be released into target cells through fusion of STB-EVs with distant cells. This ability of STB-EVs further implicates them as potential pathogenic factors in PE. However, the transcriptome of the subtypes of STB-EVs has not been thoroughly explored. Previous studies have identified differentially expressed genes (DEGs) from PE placental tissue such as *Sialic acid-binding Ig-like lectin 6* (*SIGLEC6*), *Vascular endothelial growth factor receptor 1* (*VEGFR1*), *Adrenomedullin* (*ADM*), *Pappalysin-2* (*PAPP-A2*) (8, 9), *Basic Helix-Loop-Helix Family Member E40* (*BHLHE40*), *Divergent-Paired Related Homeobox* (*DPRX*), and *HtrA Serine Peptidase 4* (*HTRA4*) (10) compared to normal placental tissue. However, none to the best of our knowledge have explored this in the context of STB-EVs between PE and normal pregnancy (NP).

We hypothesized that (1) the transcriptome of the placental tissue and STB-EVs are different between PE and NP and (2) analyzing these different sample subtypes would allow for more comprehensive and holistic profiling. We believe this strategy increases the probability of detecting relevant biomarkers and mechanistic pathways in PE. We performed RNA-sequencing (RNA-seq) on placental tissue, medium/large, and small STB-EVs from PE and NP. Our analysis revealed novel biomarkers and new insights into the possible mechanisms of preeclampsia. This knowledge may help inform future extracellular vesicle–based diagnostic tests, mechanistic experiments, and, ultimately, the development of new therapies.

## Materials and methods

Full details of the Materials and Methods section including the major resource table and research resource identifiers (RRID) are summarized in the Supplementary Material.

### Ethics approval and patient information

We obtained ethical approval from the Central Oxfordshire Research Ethics Committee C (REFS 07/H0607/74 and 07/H0606/148). We obtained written informed consent from pregnant women undergoing elective cesarean sections before labor onset at the Women's Centre, John Radcliffe Hospital, Oxford. Placentas from normal (NP, *n* = 12) and preeclamptic (PE, *n* = 12) pregnancies [for both the discovery (total *n* of 12) and the validation cohort (total *n* of 12)] were collected and perfused within 10 min of delivery. We defined NP as singleton pregnancy with no history of preeclampsia, hypertensive disorders, or other complications in pregnancy. Patients with preeclampsia were defined as the co-occurrence of *de novo* hypertension (blood pressure > 140/90 mmHg) and proteinuria (protein/creatinine ratio ≥ 30 mg/mmol) after week 20 of gestation according to the International Society for the Study of Hypertension in Pregnancy criteria (2). All PE patients used in this study were of early-onset PE (diagnosed before 34 weeks gestation).

### Enrichment of STB-EVs by placental dual-lobe perfusion and serial ultracentrifugation

We have previously published our protocol of STB-EVs isolation through *ex vivo* dual-lobe placental perfusion (Dragovic et al., 2015). Briefly, we identified a suitable cotyledon (devoid of calcifications, ischemia, or rupture) and cannulated a placental artery and vein perfusing the placenta for 3 h at a 4–5 ml/min flow rate to obtain placenta perfusate. The placenta perfusate was centrifuged twice at 1,500 g for 10 min at 4 °C (Beckman Coulter Avanti J-20XP centrifuge using a Beckman Coulter JS-5.3 swing-out rotor) to remove cell debris. The supernatant was carefully pooled and spun at 10,000 g (10 K) in a swing bucket centrifuge (Beckman L80 ultracentrifuge and Sorvall TST28.39 swing-out rotor) at 40 °C for 30 min. The 10 K STB-EV pellet was washed with filtered phosphate-buffered saline (fPBS) followed by resuspension of the 10 K STB-EV pellets in fPBS. An aliquot of the resuspended pellets was analyzed to identify and characterize STB-EVs, while the rest were aliquoted to obtain a protein concentration around 2–5 µg/µl [measured using a Pierce bicinchoninic acid (BCA) protein assay] and immediately stored at −80 °C. The post-10 K supernatant was filtered through a 0.22-µm Millipore Stericup filtration device, then spun at 150,000*g* for 2 h (Beckman L80 ultracentrifuge with a Sorvall TST28.39 swing-out rotor), and the 150 K STB-EV pellets were washed, resuspended in fPBS, and aliquoted like the 10 K STB-EV pellets. This working stock was used for subsequent analysis. Our STB-EV enrichment and categorization process has been deposited on EV-Track [(http://www.EVTRACK.org), EV-TRACK ID: EV210382] with a score of 78% correlating with excellent enrichment and categorization.

### Transmission electron microscopy

STB-EV pellets were diluted with fPBS to achieve an STB-EV solution with concentrations between 0.1 and 0.3 µg/µl. Ten microliters of the STB-EV pellet solution were applied to freshly glowing discharged carbon formvar 300 mesh copper grids for 2 min, blotted with filter paper, and stained with 2% uranyl acetate for 10 s and air-dried. STB-EV pellets on the grid were negatively stained to enhance the contrast between STB-EVs pellets and the background. The grids were imaged using an FEI Tecnai 12 TEM at 120 kV with a Gatan OneView CMOS camera.

### Flow cytometry

A BD LSRII flow cytometer (BD Biosciences) with blue, violet, and red lasers was used for all sample analyses. Daily quality control (QC) was run using CS&T beads (BD Biosciences). Photomultiplier tube (PMT) voltage determined by CS&T run was applied to all fluorescent detectors with exception for the side scatter (SSC), which was determined by Apogee Mix (1493, Apogee Flow System, UK). The SSC PMT voltage that triggered 0.59 µm silica beads and above was applied to all 10 K STB-EV pellets and analyzed. An SSC threshold of 200 was applied to remove the background noise below 0.59 µm silica beads. A flow rate of 10 µl/min was achieved using the TruCount beads (BD Biosciences). For sample staining, 90 ml of 10 K STB-EV pellet were incubated with 10 ml of Fc receptor blocker (Miltenyl, UK) for 10 min at 4 °C and then stained with phycoerythrin-conjugated placental alkaline phosphatase (PLAP) (for syncytiotrophoblast origin), PE Vio770 conjugated anti classical HLA class I and II [to exclude co-isolated non-placenta EVs and white blood cell (WBC) EV co-isolation], Pacific blue conjugated CD41 (to identify co-isolated platelet EVs), and CD235a [to identify co-isolated red blood cell (RBC) EVs] for 10 min at room temperature in the dark. Stained samples were transferred to an Ultrafree 0.2 µm filter unit (Millipore) and centrifuged at 800*g* for 3 min to remove unbound antibodies and EVs smaller than the filter pore size. Ninety microliters of fPBS were used to recover 10 K STB-EVs retained on the filter membrane. The recovered 10 K STB-EVs were further stained with BODIPY FL N-(2-aminoethyl)-maleimide (505/513 nm) (Molecular Probes) at a final concentration of 0.5 nM in the dark at room temperature for 10 min before samples were diluted to 500 ml and analyzed on the flow cytometer to check for the event rate. When necessary, dilutions were made to achieve an event rate of ≤400 counts/s and to reduce swarming. 10 K STB-EV pellets were analyzed at 10 µl/min for 10 min, and a total of 100 µl diluted samples were analyzed for each sample. Fluorescence minus one (FMO-1) for each fluorochrome and stained samples re-acquired after 2% Nonidet P-40 (NP-40) (Sigma) treatment were used as controls. Data and figures generated were generated with the Flowjo software version 10 (Tree Star Inc., Ashland, OR, USA).

### Nanoparticle tracking analysis

We further characterized the 10 K and 150 K STB-EV pellets by nanoparticle tracking analysis (NTA) [NanoSight NS500 instrument equipped with a 405 nm laser (Malvern, UK), sCMOS camera and NTA software version 2.3, Build 0033 (Malvern, UK)]. Before sample analysis, instrument performance was checked with silica 100 nm microspheres (Polysciences, Inc.). The 10 K and 150 K STB-EV pellets were individually diluted in fPBS to a range of 1/100,000. The samples were automatically injected into the sample chamber with a 1 ml syringe with the following script used for EV measurements: prime, delay 5, capture 60, repeat 4. Images of the analyzed samples were captured on camera at level 12 (Camera shutter speed: 15 ms and Camera gain: 350) and NTA post-acquisition settings were optimized and kept constant between samples. Each video recording was analyzed to infer STB-EVs size and concentration profile.

### Western blot analysis

We performed Western blots (WBs) on placental lysates (PL) and STB-EVs to further characterize and immune-phenotype. All STB-EV pellets were probed with PLAP (for syncytiotrophoblast origin), CD63 and ALIX (to confirm the presence of extracellular vesicles), and Cytochrome C (as a negative EV marker) as recommended by the International Society for Extracellular Vesicles (ISEV) (11). Following characterization and identification of extracellular vesicles in 10 K and 150 K STB-EV pellets, we renamed them to medium/large (m/l) and small (s) STB-EVs, respectively.

### RNA-sequencing library preparation and sequencing

For sequencing, RNA extraction and sample preparations were performed for placenta tissue (discovery cohort-6 NP, 6 PE) with the RNeasy mini kit and STB-EVs (6 NP, 6 PE) with the miRCURY™ RNA isolation kit for biofluids (Exiqon Services, Denmark) based on the manufacturer's protocol. The samples were sent to the Wellcome Centre for Human Genetics (WCHG) for sequencing using the standard Illumina protocol. Details can be found in the Supplementary Material.

### Bioinformatics and statistical analysis of messenger RNA sequences

We performed bioinformatics analysis using Galaxy (https://usegalaxy.org/). We used FastQC (Galaxy Version 0.72 + galaxy1) to obtain overall QC metrics and MultiQC (Galaxy Version 1.9 + galaxy1) to amalgamate the QC metrics before and after trimming of adapters [trimmomatic (Galaxy Version 0.38.0)]. Alignment was referenced to reference Homo Sapiens build 38 (hg38) genomes obtained from Ensembl with HISAT 2 (Galaxy Version 2.2.1 + galaxy0). For each sample, featureCounts (Galaxy Version 2.0.1 + galaxy1) was used to quantify genes based on reads that mapped to the provided hg38 genome. A count matrix was generated with the Column Join on Collection (Galaxy Version 0.0.3) tool. Differential expression analysis was done with the DESeq2 package (v.1.32.0 in R, v.4.0.5). The reported *p*-values were adjusted for multiple testing using the Benjamini–Hochberg correction reported as false-discovery rate. An adjusted *p*-value <0.05 was taken as significant. Functional annotation was performed with ClusterProfiler [for Gene Ontology (GO) and KEGG] and signaling pathway impact analysis (SPIA) with our transcriptome set as the background. The raw fastQ files and the processed file have been deposited in NCBI and can be accessed with the following ID: GSE190973.

### Criteria for genes selection for validation

We chose a selected group of differentially carried genes (DCGs) between NP and PE to validate based on (1) fold change {genes with a high fold change [log FC ≥ (±)1] and adjusted *p*-value ≤ 10–5}, (2) presence in at least two of the three sample types (placenta lysate, m/lSTB-EVs, and sSTB-EVs), (3) placenta specificity or enrichment, and (4) previous molecules investigated by the group. Based on expression data at the protein and RNA level from the human protein atlas (12), genes are said to be placenta specific if they are expressed only in the placenta, while genes are placenta enriched if the placenta is one of the five with the highest expression.

### Quantitative polymerase chain reaction for mRNA validation

Following standard protocol, the high-capacity cDNA reverse transcription kit (Applied Biosystems, USA) was used for reverse transcription of the validation cohort [*n* = 6 (PE), *n* = 6 (NP)]. Quantitative polymerase chain reaction (qPCR) was performed on Quant Studio™ 3 real-time PCR systems (QuantStudio™ design and analysis software), MicroAmp™ Optical 96-well reaction plate (N8010560), and optical adhesive film kit (4313663), with the hydrolysis probe-based TaqMan (R) gene expression assay (Applied Biosystems, USA). The following settings were used: hold 50 °C for 2 min, hold at 95 °C for 20 s, followed by 40 cycles of denaturation at 95 °C for 1 s and annealing/extension at 60 °C for 20 s. Cq values were generated automatically by the Quant Studio Design and Analysis desktop software. The geometric mean of *YWHAZ* and *SDHA* were used as the internal reference genes for the normalization of all qPCR data. We analyzed the qPCR data and calculated fold changes using the 2^−ΔΔCt^ method (13). Data were expressed as fold change, and standard error is denoted as error bars with GraphPad Prism software (version 9). Statistical testing was made on the ΔCt values using a one-tailed Student *t*-test, and significance was set at *p* < 0.05. All gene expression assays, their corresponding assay IDs, and all other details used in this study are listed in the Supplementary Material.

## Results

### Patient demographics and clinical characteristics

There were no significant differences in maternal age, body mass index, and the gender of the neonates (Table 1). The average systolic (178.83 mmHg) and diastolic (109.17 mmHg) blood pressures were significantly higher among the PE cohort (*p* < 0.001). Likewise, there was a significant difference in proteinuria (PE = 2.58; NP = 0 pluses on urine dipstick; *p* < 0.001) and gestational age at delivery (PE = 32.00 weeks gestation, NP = 39.17 weeks gestation; *p* = 0.001) in PE compared to NP. Finally, PE neonates were more likely to be growth restricted (100%, and 0%; *p* = 0.004) with an average birth weight of 1,515.83 g compared to 3,912.50 g in normal neonates (*p* < 0.001).

**Table 1:** General characteristics of the transcriptomic cohort study population.

Characteristics	Normal pregnancy	Preeclampsia	*p*-value
Sample size^a^	6	6	
Maternal age, years [mean (SD)]	34.50 ± 5.39	36.33 ± 4.13	0.524
Body mass index, kg/m^2^ [mean (SD)]	29.92 ± 9.10	31.25 ± 11.12	0.825
Systolic blood pressure, mmHg [mean (SD)]	129.50 ± 4.93	178.83 ± 12.56	<0.001
Diastolic blood pressure, mmHg [mean (SD)]	67.00 ± 6.20	109.17 ± 9.85	<0.001
Proteinuria plus(es) [mean (SD)]	0	2.58 ± 1.20	<0.001
Gestational age at delivery in weeks [mean (SD)]	39.17 ± 0.98	32.00 ± 3.52	0.001
Birth weight, g [mean (SD)]	3,912.50 ± 730.40	1,515.83 ± 600.57	<0.001
Intrauterine growth restriction (IUGR) = Yes (%)^−^	0 (0%)	6 (100%)	0.004
Male newborn gender (%)	2 (33.3%)	2 (33.3%)	1.000

^a^ Sample size for the transcriptomics cohort [PL (NP = 6, PE = 6) and STB-EVs (NP = 6, PE = 6)]. qPCR confirmation for PL was performed on the same cohort [PL (NP = 6, PE = 6)], while STB-EV qPCR validation was performed on a different cohort (NP = 6, PE = 6)—those patient characteristics are available in the Supplementary Material.

### Characterization of STB-EVs by TEM, NTA, flow cytometry, and WB

**Figure 1:**
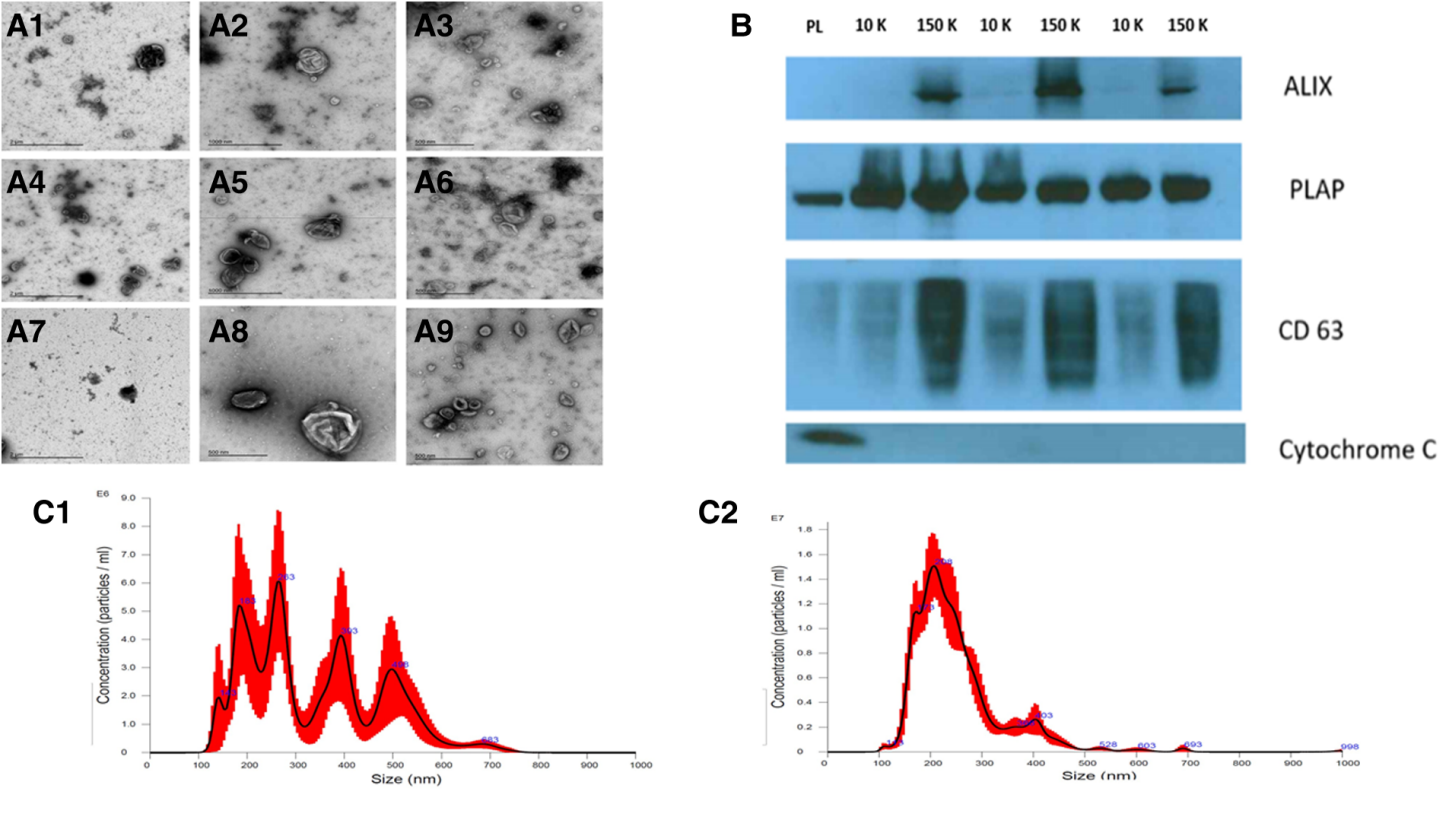
STB-EV characterization. (**A**) displays the representative TEM images with a wide view of samples (**A1, A4, A7**), m/lSTB-EVs (**A2, A5, A8**) displaying heterogeneity of vesicle sizes, and sSTB-EVs (**A3, A6, A9**) displaying typical cup-shaped morphology. (**B**) is a Western blot of placenta homogenate (PL), m/lSTB-EVs (10 K), and sSTB-EVs (150 K) showing positivity for PLAP in all samples confirming placental origin. sSTB-EVs express enhanced levels of ALIX and CD63, which are known sSTB-EV markers. The absence of cytochrome C in the STB-EV population confirms no co-isolation with apoptotic bodies. (**C**) shows the NTA results of m/lSTB-EVs (**C1**), which show a broad size distribution, and sSTB-EVs (**C2**), which display a more homogeneous size.

We characterized our sample preparations with transmission electron microscopy (TEM), NTA, flow cytometry (FC), and WB after the isolation and enrichment of STB-EVs. TEM (Figure 1A) showed the typical cup-shaped morphology of extracellular vesicles. In particular, the 10 K STB-EV pellet (Figures 1A2, 1A5, 1A8) showed a size heterogeneity characteristic of m/lSTB-EVs (221–1,000 nm) and the 150 K STB-EV pellet (Figures 1A3, 1A6, 1A9) showed a homogeneous EVs size profile (≤220 nm). This finding was also replicated by NTA (Figure 1C). The 10 K STB-EV (Figure 1C1) pellets had a modal size of 479.4 ± 145.6 nm, while the 150 K STB-EV (Figure 1C2) pellets had a smaller modal size (205.8 ± 67.7 nm). The post-10 K and post-150 K pellets are hereafter renamed m/lSTB-EVs and sSTB-EVs, respectively. WB (Figure 1B) detected PLAP (66 KDa), tetraspanins [CD63 (30–65 KDa)], and endosomal trafficking proteins [ALIX (95 KDa)]. The non-EV marker cytochrome C (12 KDa) was detected in the placenta lysate but not the STB-EV fractions. Notably, TSG101 and ALIX were more prominent in the sSTB-EV fractions than the m/lSTB-EV fractions and placenta lysate.

Flow cytometry (Supplementary Figure S1) was only performed on the m/lSTB-EVs due to the size detection limit of flow cytometry, which does not permit interrogation of small STB-EVs. We found that 85 ± 8.3% (Supplementary Figure S1C) of qualified events were negative for the following lineage markers [CD41 (platelets) and CD235a (red blood cells), and HLA-I and II (white blood cells)]. Also, 95 ± 1.2% of the EVs negative for non-placental markers (listed above) were BODIPY FL N-(2-aminoethyl)-maleimide (bioM) and PLAP double-positive (Supplementary Figure S1D). PLAP is a specific marker of syncytiotrophoblast and bioM a marker of EVs, so this analysis confirmed that most of the post-10 K samples, within the detection size range, were of placental origin. NP-40 (detergent) treatment confirmed that our samples were largely vesicular since only 0.1 ± 0.12% BODIPY FL N-(2-aminoethyl)-maleimide and PLAP double-positive events were detected (a reduction of 99%) after treatment with detergent (Supplementary Figure S1E).

### DCGs in placenta homogenate, m/lSTB-EVs, and sSTB-EVs between PE and NP

**Figure 2:**
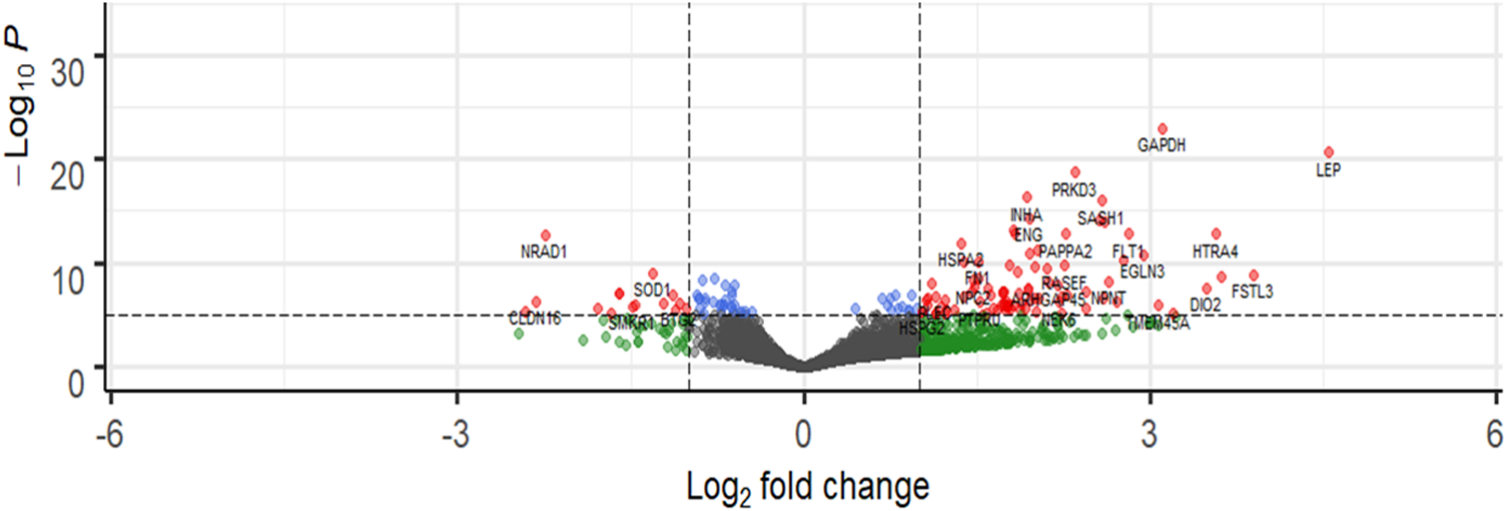
Representative volcano plot showing differentially abundant genes in m/lSTB-EVs. The most significantly upregulated genes are displayed in red on the right, while the most significantly downregulated genes are displayed in red on the left. Volcano plots for placenta and sSTV-EVs are in the Supplementary Material. −Log_10_P refers to the negative logarithm of the adjusted *p*-value.

Transcriptomic analysis was performed on the discovery cohort [*n* = 6 (PE), *n* = 66 (NP)]. Comparison between PE and NP placental tissue revealed 580 upregulated and 563 downregulated (total) genes [adjusted *p*-value of <10^−5^ (Supplementary Figure S3)], while in m/lSTB-EVs, 1,128 were upregulated and 833 were downregulated [adjusted *p*-value of <10^−5^ (Figure 2)]. In sSTB-EVs, 232 were upregulated and 106 were downregulated (adjusted *p*-value of <10^−5^ (Supplementary Figure S4)]. We noted that 25 downregulated genes and 120 upregulated genes were common to all three sample types.

### qPCR validation of selected genes

**Figure 3:**
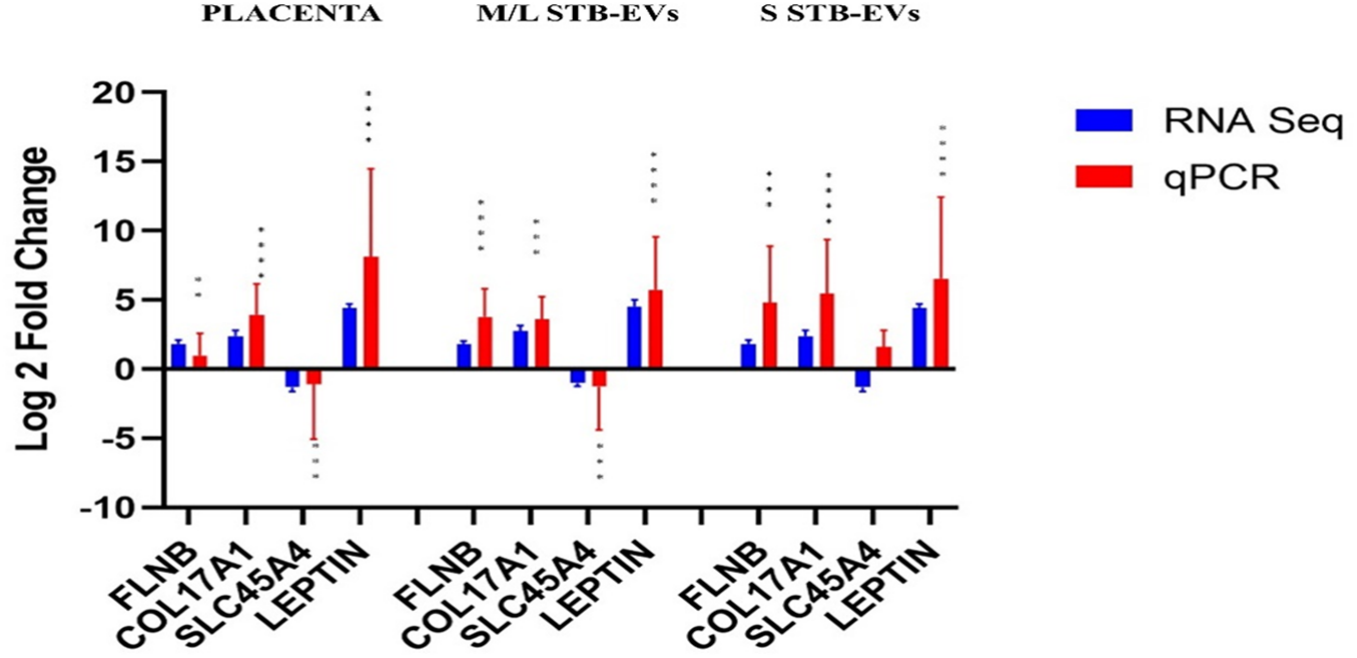
Quantitative PCR validation of selected differentially expressed/carried genes *LEP*, *COL17A1*, *SLC45A4*, *FLNB*, and *ENG* in the placenta, m/lSTB-EVs, and sSTB-EVs. Data are visualized as mean fold change, and error bars represent standard error. *p < 0.05; **p < 0.01; ***p < 0.001; and ****p < 0.0001. Sample size (*n*) = 6 (NP) and *n* = 6 (PE).

**Figure 4:**
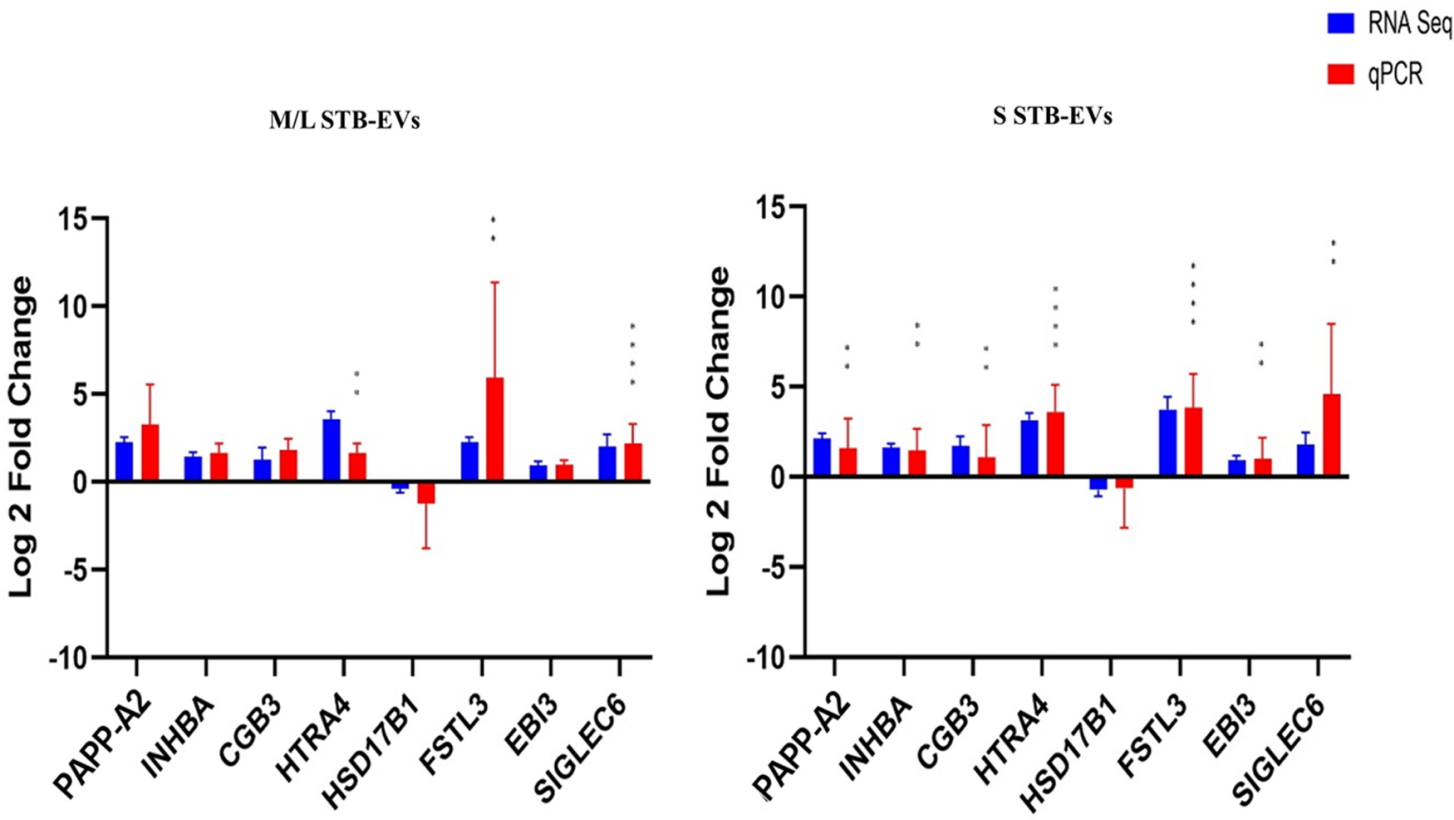
Quantitative PCR validation of placenta-specific genes: *PAPP-A2*, *INHBA*, *SIGLEC6*, *HTRA4*, *EBI3*, *FSTL3*, *HSD17B1*, *CGB3*, and *ARMS2* in m/lSTB-EVs and sSTB-EVs based on qPCR. Data are visualized as mean fold change, and error bars represent standard error. *p < 0.05; **p < 0.01; ***p < 0.001; and ****p < 0.0001. Sample size (*n*) = 6 (NP) and *n* = 6 (PE).

Quantitative PCR was performed on a different subset of STB-EVs, the validation cohort [*n* = 6 (PE), *n* = 6 (NP)]. As discussed earlier in the Materials and Methods section, we identified the DCGs that were common between all three sample types (common DCGs), and of those, we selected a subset that were placenta enriched or specific based on the criteria mentioned in the section. We selected *HTRA4*, *PAPP-A2*, *EBI3*, *HSD17B1*, *FSTL3*, *INHBA*, *SIGLEC6*, and *CGB3*. We performed qPCR analysis of these genes in both m/lSTB-EVs and sSTB-EVs. As a proof of concept, four of these genes known to be altered in PE in placental tissues (*LEP*, *COL17A1*, *SLC45A4*, and *FLNB*) were analyzed in the placenta, and we confirmed that these genes were indeed significantly altered in PE (Figure 3). We next moved on to the species of interest and showed that in the m/lSTB-EVs (Figures 3, 4), *LEP*, *SIGLEC6*, *FLNB*, *COL17A1*, *SLC45A4*, *FSTL3*, and *HTRA4* were significantly different in PE compared to NP.

In sSTB-EVs (Figures 3, 4), all the selected genes (except for *SLC45A4* and *HSD17B1*) were significantly different. Across the three sample types, *LEP*, *COL17A1*, and *FLNB* were all significantly different between PE and NP, while *SIGLEC6*, *FSTL3*, and *HTRA4* were significantly different in both m/lSTB-EVs and sSTB-EVs.

### Functional enrichment analysis and SPIA of DEGs in PE

To understand the role of these genes and perhaps STB-EVs in the pathophysiology of preeclampsia, we performed a functional enrichment analysis (with overrepresentation analysis) on the list of DCGs (between NP and PE) for placenta tissue, m/lSTB-EVs, and sSTB-EVs and identified functional processes that overlap among the three sample subtypes. There were 32 similar biological processes between the placenta and sSTB-EVs (see full list in Supplementary Table S4). One biological process was similar between the placenta and m/lSTB-EVs (platelet degranulation), two biological processes (cell adhesion molecule and integrin-binding) were common to both placenta and sSTB-EVs, and no biological process was common to all sample subtypes.

When analyzing KEGG pathways, focal adhesion was overrepresented among all three sample types, while in the HIF-1 signaling pathway, proteoglycans in cancer and central carbon metabolism in cancer were overrepresented in both placental tissue and sSTB-EVs.

Signaling pathway impact analysis of the DEGs in placental tissue homogenate showed neuroactive ligand–receptor interaction, extracellular matrix (ECM)–receptor interaction, focal adhesion, amebiasis, and gap junction as the most overrepresented. Of these five, all were inhibited except the neuroactive ligand–receptor interaction, which was activated. In contrast, the same analysis on m/lSTB-EVs revealed two significantly dysregulated pathways, focal adhesion and cytokine–cytokine interaction pathways, both of which were activated. Similarly, in sSTB-EVs, three pathways, adipocytokine, focal adhesion, and type II diabetes mellitus (DM), were significantly activated. Among all three sample types, focal adhesion was common to all.

## Discussion

In recent years, EVs have gained interest, particularly their role in the context of PE, their potential to affect functional changes in organs distant from the placenta, and their ability to act as circulating liquid biopsies, revealing data about the donor cells in real time. Our comprehensive analysis has unveiled a distinct transcriptomic signature in PE-associated STB-EV compared to those in NP. Among the identified and validated STB-EV mRNAs are *FLNB*, *COL17A1*, *SLC45A4*, *LEP*, *HTRA4*, *PAPP-A2*, *EBI3*, *HSD17B1*, *FSTL3*, *INHBA*, *SIGLEC6*, and *CGB3*. Exploration of these differentially abundant mRNAs as circulating biomarkers may help in the early identification of PE, providing valuable insights into potential pathological mechanisms.

Some of the findings of this study, such as *FLT1*, *LEP* (14), *ENG*, *PAPP-A2* (15), *FSTL3, PRG2* (16), *INHBA* (17), which have been well described in the placenta, were not previously associated with STB-EVs. These STB-EV-associated mRNA may account for some circulating mRNAs such as *PAPP-A2*, *LEP*, and *HTRA4* (18) detected in increased amounts in plasma of PE patients. To validate their biomarker potential, we opted to validate the following genes in both m/lSTB-EVs and sSTB-EVs: *FLNB*, *COL17A1*, *SLC45A4*, *LEP*, *ENG*, *HTRA4*, *PAPP-A2*, *EBI3*, *HSD17B1*, *ARMS2*, *FSTL3*, *INHBA*, *SIGLEC6*, and *CGB3*, since these fulfilled the criteria for biomarkers as outlined. We discovered that on m/lSTB-EVs, *FLNB*, *COL17A1*, *SLC45A4*, *LEP*, *FSTL3*, *HTRA4*, and *SIGLEC6* were significantly differentially expressed between PE and normal. Equally interestingly, in sSTB-EVs, we found that except for *HSD17B1*, all the other genes were differentially carried between PE and normal.

*LEP* was the most significantly elevated gene in placental tissue, m/lSTB-EV, and sSTB-EVs. The discovery of *LEP* in STB-EVs is novel. While the potential of LEP protein, rather than its mRNA, as a potential biomarker has been suggested previously, its presence in STB-EVs adds a new dimension (19). *LEP* plays a role in endocrine functions, reproduction, immune function, and is produced by the placenta, aiding implantation and regulating placental growth (20). Leptin dysregulation has been linked to pregnancy complications with higher circulating protein levels associated with PE (21). However, it is crucial to recognize that the exact role of leptin in preeclampsia's development remains incompletely understood. One review article suggests that intermittent hypoxia, a consequence of sleep-disordered breathing, may impact leptin levels in pregnant women, rather than preeclampsia's pathogenesis (22). Another study showed that *LEP*-induced endothelial dysfunction, a possible cause of preeclampsia, might happen even if there are no other health problems related to obesity (23). The significance of finding elevated *LEP* mRNA in STB-EVs in the context of PE extends beyond a mere correlation. It suggests a potential mechanism by which STB-EVs, laden with *LEP* mRNA, may serve as messengers, transporting genetic information to organs and tissues far from the placenta and thus potentially linking *LEP* to PE. This genetic cargo could contribute to the complex clinical manifestations of PE, a condition marked by systemic vascular dysfunction and various organ involvements.

Similarly, we found significantly elevated *COL17A1* in the placenta, m/lSTB-EVs, and sSTB-EVs. *COL17A1* is a collagen transmembrane protein involved in extracellular matrix–receptor interactions (24). It suggests a potential link between the deregulation of extracellular matrix interactions and the development of PE. Moreover, our findings align with prior research that *COL17A1* is differentially expressed in PE (25). COL17A1 protein can be detected in urine samples from pregnant patients, but differential expression in terms of PE compared to NP has not been thoroughly investigated (26). It would be intriguing to explore if the abundance of circulating *COL17A1* in PE is translated to the urine. In contrast, we found that *SLC45A4* mRNA was significantly downregulated in both types of vesicles. *SLC45A4* protein is involved in promoting glycolysis and autophagy prevention (27), two processes found to be abnormal in PE (28). The role of SLC45A4 in PE remains undiscovered and would form the basis of further research from our group.

Filamin B (*FLNB*) is a cytoskeletal protein that regulates various cellular processes, including actin organization, cell adhesion, migration, and signal transduction. The precise pathological role of *FLNB* in PE remains incompletely elucidated. However, emerging evidence indicates that *FLNB* may contribute to the dysregulation of cellular pathways implicated in preeclampsia (29). Transcriptomic profiling has revealed aberrant *FLNB* expression and alternative splicing patterns in preeclamptic placentas compared to normotensive controls (29). *FLNB* has been previously reported to be downregulated in PE placentae at the mRNA and protein level (30, 31). This is in contrast with our study, which showed that the *FLNB* gene was significantly elevated in the placenta and in m/lSTB-EVs and sSTB-EVs. It is difficult to identify the exact reason for the variance, but the choice of the housekeeping gene may be a possible explanation. Wei et al. (30) used *β-actin*, which has been observed to be variable and a potential confounder in other disease states or experimental conditions (32). We used the geometric mean of *YWHAZ* and *SDHA*, both previously shown to be stably expressed (33). The multifunctional nature of *FLNB* suggests potential interactions with established preeclampsia factors, including angiogenic/antiangiogenic proteins, insulin signaling defects, and immune dysregulation. Further mechanistic studies defining changes in *FLNB* expression, post-translational modification, and protein interactions in the context of preeclampsia will clarify its contributions to this complex hypertensive disorder of pregnancy. While the precise molecular mechanisms in PE are unknown, *FLNB* participates in preeclampsia-related pathways, including transcriptional regulation, alternative splicing, and apoptosis regulation in trophoblastic cells (29). Elucidating the functional impact of aberrant *FLNB* expression and activity in preeclamptic cells remains an active area of investigation.

Our analysis also showed that *SIGLEC6* was differentially present in both subsets of STB-EVs. Our group had previously reported the presence of *SIGLEC6* in higher concentrations in PE placentas compared to NP (34). *SIGLEC6* regulates immune cells by interacting with sialic acid on these cells and inhibiting cellular activation and clonal expansion (35) through cytosolic tyrosine-based regulatory motifs (36). Quantitative gene expression analyses have revealed the upregulation of *SIGLEC6* transcript and protein levels in preeclamptic placentae compared to normal controls (37). The distinct expression pattern of *SIGLEC6* in the human placenta, coupled with its known roles in regulating immune cell interactions and invasion processes, suggests that it may contribute to the aberrant immune activation and shallow trophoblast invasion characteristic of preeclampsia (38). Interestingly, *SIGLEC6* appears to be differentially expressed in parallel with other genes implicated in related disorders like the HELLP syndrome that may denote a common pathway in hypertensive diseases of pregnancy (39). Overall, these findings position *SIGLEC6* as a putative regulator of immune and trophoblastic functions relevant to preeclampsia pathogenesis. Further mechanistic studies examining *SIGLEC6*'s specific molecular interactions and signaling effects in placental cells may provide insights into its utility as a biomarker or therapeutic target for PE.

We also identified *FSTL3* to be differentially abundant in PE compared to NP. *FSTL3* glycoprotein is localized to the decidua and has been well-documented to be present in abundance in PE placenta and plasma samples (40–42). Quantitative gene and protein expression analyses have detected the upregulation of FSTL3 in preeclamptic placentae and maternal circulation compared to normotensive controls (43). *In vitro*, *FSTL3* is transcriptionally induced by hypoxic stress in cultured trophoblasts, consistent with deficient uteroplacental perfusion as an inciting event in preeclampsia pathogenesis (44, 45). Functional investigations demonstrate that *FSTL3* knockdown promotes apoptosis and inhibits proliferation, migration, invasion, and lipid accumulation in trophoblasts—cellular phenotypes that mirror the shallow trophoblast invasion and abnormal placentation observed in preeclampsia (43). While the predictive value of first trimester circulating *FSTL3* levels remains inconclusive, the collective data support a contributory role for *FSTL3* in preeclampsia pathogenesis (46). Our analysis demonstrates that this elevation in *FSTL3* is also mirrored at the mRNA level in STB-EVs. However, considerable knowledge gaps remain regarding the upstream factors regulating aberrant *FSTL3* expression and the downstream signaling pathways through which it exerts effects in trophoblastic cells. Further elucidating the molecular interactions of *FSTL3* and integrating these findings through systems biology approaches will provide insights into its utility as a biomarker or therapeutic target in preeclampsia.

Human high-temperature requirement serine peptidase A4 (*HTRA4*) is a placental-specific protease emerging as a pathogenic factor and biomarker in preeclampsia (47). *HTRA4* was found to be differentially carried in PE STB-EVs in our analysis. *HTRA4* is a placental-specific serine protease that inhibits cell cycle progression and cell differentiation to endothelial cells (48). *HTRA4* may be responsible for endothelial cell dysregulation by disrupting endothelial tube formation and increasing endothelial cell permeability (49). Mechanistically, *HTRA4* has been shown to degrade extracellular matrix proteins like fibronectin and cleave endothelial junction protein vascular endothelial cadherin—effects that may impair trophoblast invasion and maternal vascular integrity in preeclampsia (50, 51). *In vitro*, *HTRA4* attenuates migration and proliferation of trophoblastic cells, while hypoxic stress potently induces *HTRA4* expression (52). Moreover, *HTRA4* can suppress syncytin-1-mediated trophoblast fusion (51, 53). These combined findings indicate that *HTRA4* may contribute to insufficient trophoblast invasion and placental dysfunction in preeclampsia. In parallel, the increased expression of the related protease *HTRA1* in preeclamptic placentae underscores the pathophysiologic significance of dysregulated *HTRA* family members in this hypertensive pregnancy disorder (52). Further elucidating the upstream regulation and precise molecular interactions of *HTRA4* in placental cells will provide greater insight into its role as a candidate driver and biomarker in preeclampsia pathogenesis.

We also attempted to provide mechanistic insights into the roles of these differentially carried genes in the pathogenesis of PE. We identified hypoxia-inducible factor 1 (*HIF 1*) *pathway*, *blood vessel development* (15), *adipocyte regulation*, *retinoic acid regulation*, *hypoxic signaling*, *associated cytokine pathways* (54), *PI3/Akt signaling*, and *pathways in cancer* (55) as pathways that are potentially contributory. The *HIF 1* signaling pathway is essential for regulating diverse biological processes including angiogenesis, adipogenesis, and the cellular response to hypoxia. *HIF 1* is a heterodimeric transcription factor composed of *HIF 1α* and *HIF 1β* subunits that are stabilized and activated under low oxygen conditions (56). In the context of blood vessel development, *HIF 1* cooperates with the E2F7 and E2F8 transcription factors to induce vascular endothelial growth factor A (*VEGFA*) expression, promoting angiogenesis (57). In addition, the adipokine leptin has been shown to have pro-angiogenic effects on endothelial cells, stimulating their proliferation, migration, and tube formation through *HIF 1*-dependent upregulation of *VEGFA* (58). Retinoic acid, a metabolite of vitamin A, also plays a key role in vascular development by modulating the expression of angiogenic regulators like *VEGFA* (59). Dysregulation of retinoic acid and its target genes has been observed in vascular malformations such as cerebral arteriovenous malformations (59). In addition to its angiogenic functions, the *HIF 1* pathway has been shown to regulate adipocyte biology. *HIF 1* activation in adipocytes induces the expression of genes involved in adipogenesis and lipid metabolism (56). The adipokine leptin also influences adipocyte function partially through *HIF 1*-dependent mechanisms, promoting metabolism and apoptosis in peripheral adipose depots (60). At the molecular level, the *PI3K/Akt* signaling cascade is a key regulator of cell growth, survival, metabolism, and angiogenesis that crosstalk with *HIF 1* signaling (61). Aberrant activation of *HIF 1* and *PI3K/Akt* pathways synergistically reprogram cancer cell metabolism and survival in hypoxic microenvironments and may potentially perform the same regulatory role in PE (61).

The *HIF 1* and *PI3K/Akt* signaling cascades are intricately connected in regulating the cellular response to hypoxia. Under hypoxic conditions, *HIF 1α* protein stability and activity is promoted through Akt-mediated inhibition of proteasomal degradation (56). Conversely, *HIF 1* signaling induces the expression of various growth factors that feed back to activate *PI3K/Akt* signaling (62). In particular, the *PI3K/Akt* axis is known to modulate angiogenesis through effects on endothelial cell migration, proliferation, and survival (63). Similarly, retinoic acid signaling regulates the expression of pro-angiogenic factors during development (64). Given the established roles of *PI3K/Akt* and retinoic acid and other identified pathways in vascular development, it is plausible that dysregulation of these pathways may disrupt the coordinated placental angiogenesis required for normal pregnancy. This crosstalk exemplifies the complex network of interactions involved in maintaining oxygen homeostasis. While less studied in the context of preeclampsia, aberrant regulation of these interconnected pathways may contribute to the defective placental angiogenesis and vascularization underlying this hypertensive disorder of pregnancy.

We also found several less well-characterized and potentially relevant pathways such as *processes involved with protein catabolism and degradation*, *insulin and other growth factor receptor signaling pathways*, *nervous system development*, *ion channel activities*, and *transcription repression*, among others. Interestingly, our signaling pathway impact analysis revealed intriguing patterns. While the *focal adhesion pathway* was inhibited in the placenta, it was activated in both STB-EV subtypes. *Focal adhesion* is a critical step in cell and ECM interactions and perhaps interactions between the trophoblast and the ECM and is crucial in trophoblastic invasion and PE (65). C*ytokine–cytokine interactions* were activated in m/lSTB-EVs, while *type II DM* and *adipocytokine* pathways were activated in sSTB-EVs. The risk of PE is known to be increased by two- to fourfold among type II DM patients, and women with a history of PE are more likely to develop type II DM later in life (66). None of the patients used in this study had been diagnosed with diabetes mellitus. If they had, it may have explained why this pathway was activated in our analysis. It may be interesting to explore, in the future, if sSTB-EVs play a role in this association or if this association is due to the presence of comorbid conditions common to both PE and DM. It is reasonable to assume that the differences in the GO terms and pathways among all sample types may portray a difference in roles between the placenta, m/lSTB-EVs, and sSTB-EVs in the pathogenesis of the disease. However, we believe that these differences may be best explored in future studies.

Our findings have identified several potential STB-EV mRNA biomarkers that require external validation especially in biofluids like plasma, serum, or urine. The prospect of a qPCR biomarker panel is interesting as a qPCR panel should be cheaper and easier to implement than a quantitative protein assay. Such tests are easily scalable and if validated might even be adaptable to low-income resource settings using the loop-mediated isothermal amplification (LAMP) PCR, a PCR technique than can be performed at room temperature. Since transcriptome-level alterations precede proteome-level alterations, mRNA-based biomarkers may theoretically be better at ascertaining the risk of developing preeclampsia before the development of clinical symptoms than standard of care. We believe that mRNA-based biomarkers have the potential to revolutionize the early identification of PE. Nevertheless, their integration into clinical contexts may confront two potential obstacles. First, the diversity in mRNA expression across individuals complicates the establishment of a universally applicable diagnostic threshold. Second, the requirement for standardized procedures in sample collection, processing, and analysis presents challenges within clinical settings.

Our study is not without limitations that should be considered while interpreting the results. First, our sample size is relatively small and thus no predictive analysis could be conducted. Second, it is difficult to find a gestational age–matched control for PE, particularly the early-onset phenotype (<34 weeks). Therefore, we used term (37–41^+6^ weeks) controls. This is a standard approach for all scientists working on PE placentas. Finally, like most studies on PE, the samples are from deliveries. It is possible that these biomarkers represent differences observed at end-stage disease and would require further research to translate these markers to early developmental stages. Finally, the accuracy of the identified biomarkers may be influenced by environmental factors and comorbidities, such as diabetes, chronic kidney disease, and antiphospholipid syndrome. It is important to note that none of our patients had these comorbidities. While the impact of epigenetics is well-documented and should be taken into consideration during interpretation, accounting for all environmental factors can be a challenging task (67). We believe that this study represents an initial and reasonable step in the identification of potential biomarkers.

Notwithstanding, this study has numerous positives. One notable advantage is our comprehensive analysis of three linked sample types from the same patient. This has permitted the dissection of differences between the tissue and the EVs and the identification of some potentially exciting pathways that may form the basis of further research. These findings serve as a foundation for future research in the PE field, holding a promise for a deeper understanding of this complex pregnancy disorder and providing potential biomarkers. We believe that these candidate genes should undergo further evaluation in a more controlled experimental design, allowing for additional research and validation. Ultimately, our objective is to translate these findings into practical clinical applications.

## Conclusions

Despite intensive efforts, the pathophysiology of PE has still not been completely unraveled. In this study, we identified potential mRNA biomarkers and mechanistic gene pathways that may be important in the pathophysiology of PE and could be further explored in future studies. The potential utilization of STB-EV-based mRNA as circulating biomarkers with real-time placental and fetal information may be important in early PE diagnosis and mechanism.

## Data Availability

The datasets presented in this study can be found in online repositories. The names of the repository/repositories and accession number(s) can be found in the article/**Supplementary Material**.
