# Supplementary material for: A cross-sectional analysis of syncytiotrophoblast membrane extracellular vesicles–derived transcriptomic biomarkers in early-onset preeclampsia
Source: Front Cardiovasc Med. 2023 Nov 30;10:1291642. doi: 10.3389/fcvm.2023.1291642 (PMC10720444; doi:10.3389/fcvm.2023.1291642)
Supplement: Supplementary file 1 [file Datasheet1.docx]

Supplementary Material

***Supplemental Methods***

***Enrichment of STB-EVs by placental dual-lobe perfusion and serial ultracentrifugation***

Briefly, we identified a suitable cotyledon (devoid of calcifications, ischemia, or rupture) and cannulated a placental artery and vein perfusing the placenta for three hours at a 4-5 ml/min flow rate to obtain placenta perfusate. The placenta perfusate was centrifuged twice at 1,500 g for ten minutes at 4oC (Beckman Coulter Avanti J-20XP centrifuge using a Beckman Coulter JS-5.3 swing-out rotor) to remove cell debris. The supernatant was carefully pooled and spun at 10,000 g (10K) in a swing bucket centrifuge (Beckman L80 ultracentrifuge and Sorvall TST28.39 swing-out rotor) at 40C for 30 minutes. The 10K STB-EV pellet was washed with filtered phosphate buffer saline (fPBS) followed by resuspension of the 10K STB-EV pellets in fPBS. An aliquot of the resuspended pellets was analyzed to identify and characterize STB-EVs, while the rest were aliquoted to obtain a protein concentration around 2-5 µg/µl (measured using a Pierce bicinchoninic acid (BCA) protein assay) and immediately stored at -80oC. The post-10K supernatant was filtered through a 0.22 µm Millipore stericup filtration device, then spun at 150,000 g for 2 hours (Beckman L80 ultracentrifuge with a Sorvall TST28.39 swing-out rotor) and the 150K STB-EV pellets were washed, resuspended in fPBS and aliquoted like the 10K STB-EV pellets. This working stock was used for subsequent analysis.

***Transmission electron microscopy***

STB-EV pellets were diluted with fPBS to achieve an STB-EV solution with concentrations between 0.1-0.3 µg/µl. Ten microliter of the STB-EV pellet solution was applied to freshly glowing discharged carbon formvar 300 mesh copper grids for two minutes, blotted with filter paper, and stained with 2% uranyl acetate for ten seconds and air-dried. STB-EV pellets on the grid were negatively stained to enhance the contrast between STB-EVs pellets and the background. The grids were imaged using an FEI Tecnai 12 TEM at 120 kV with a Gatan OneView CMOS camera.

***Flow Cytometry***

A BD LSRII flow cytometer (BD Biosciences) with a blue, violet, and red laser was used for all sample analyses. Daily quality control (QC) was run using CS&T beads (BD Biosciences). Photomultiplier tube (PMT) voltage determined by CS&T run was applied to all fluorescent detectors with exception for the side scatter (SSC) which was determined by Apogee Mix (1493, Apogee Flow System, UK). SSC PMT voltage that triggered 0.59 µm silica beads and above was applied to all 10K STB-EV pellets and analyzed. An SSC threshold of 200 was applied to remove background noise below 0.59 µm silica beads. A flow rate of 10 µl/min was achieved using the TruCount beads (BD science). For sample staining, 90 ml of 10K STB-EV pellet were incubated with ten ml of Fc receptor blocker (Miltenyl, UK) for 10 minutes at 4OC and then stained with phycoerythrin (PE) conjugated PLAP (for syncytiotrophoblast origin), PE Vio770 conjugated anti classical HLA class I and II (to exclude co-isolated non-placenta EVs and white blood cell (WBC) EV co-isolation), Pacific blue conjugated CD41 (to identify co-isolated platelet EVs) and CD235a (to identify co-isolated red blood cell (RBC) EVs) for ten minutes at room temperature in the dark. Stained samples were transferred to an Ultra free 0.2 µm filter unit (Millipore) and centrifuged at 800 g for three minutes to remove unbound antibodies and EVs smaller than the filter pore size. Ninety microliters of fPBS were used to recover 10K STB-EVs retained on the filter membrane. Recovered 10K STB-EVs were further stained with BODIPY FL N-(2-aminoethyl)-maleimide [505/513 nm] (Molecular Probes) at a final concentration of 0.5 nM in the dark at room temperature for ten minutes before samples were diluted to 500 ml and analyzed on the flow cytometer to check for events rate. When necessary, dilutions were made to achieve an events rate of ≤ 400 count/second and to reduce swarming. 10K STB-EV pellets were analyzed at 10 µl/minute for ten minutes and a total of 100 µl diluted samples was analyzed for each sample. Fluorescence minus one (FMO-1) for each fluorochrome and stained samples re-acquired after 2% Nonidet P-40 (NP-40) (Sigma) treatment were used as controls. Data and figures generated were generated with the Flowjo software version 10 (Tree Star Inc., Ashland, OR).


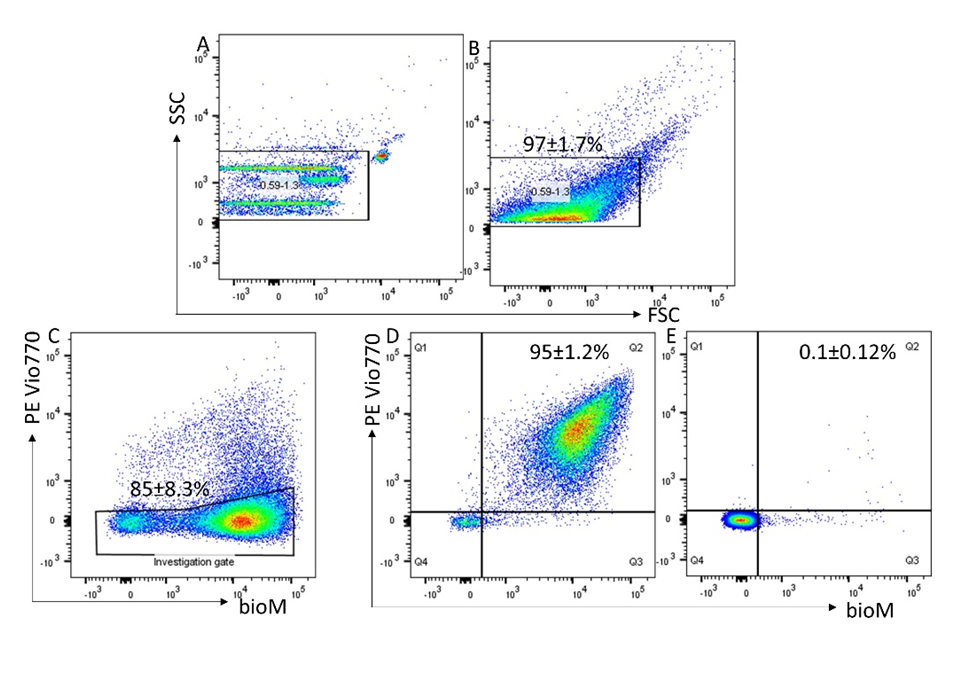


**Figure S1.** Representative flow analysis of m/lSTB-EVs enriched by placental perfusion. Apogee beads mix was used to set the flow machine’s light scatter resolution to 0.59-1.3 mm silica beads (Figure S1A); Application of SSC and FSC PMTVs determined by apogee beads mix for the analysis of M/L STB-EVs (Figure S1B). An investigation gate interrogating m/l STB- EVs which did not express the non-placental markers HLA Class 1 & II, CD41a and CD235a – thus removing co-isolated non-placental EVs (Figure S1C). STB-EVs from the investigation gate were further analyzed for expression of PLAP and staining by bioM- which stains proteins (Figure S1D). This revealed a high number (95%) of PLAP positive vesicles. These PLAP+ bioM+ double positive EVs were highly sensitive to detergent treatment (Panel E) with the reduction in PLAP+ bioM+ double positivity confirming that they are vesicular in nature.


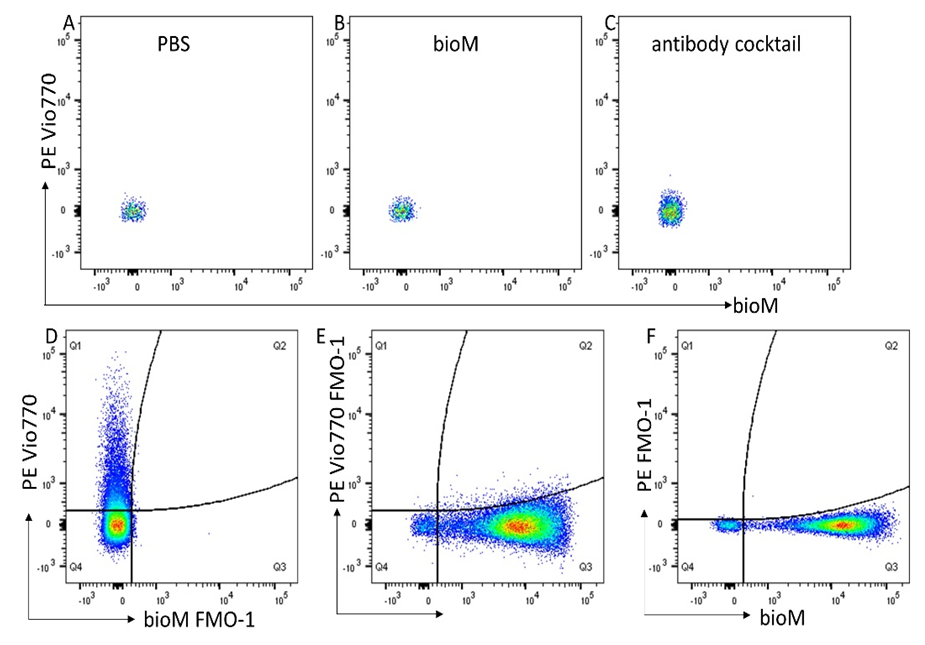


**Figure S2.** Reagent controls, 0.1mm filtered PBS (A); 0.2mm filtered bioM (B) and antibody cocktail (C) all showing no PLAP+ bioM+ double positivity. Fluorescent minus one (FMO-1) controls, bioM-1 control (D); PE Vio770-1 control (E); PE-1 control (F).

***Nanoparticle Tracking Analysis***

We further characterised the 10K and 150K STB-EV pellets by nanoparticle tracking analysis [(NTA) NanoSight NS500 instrument equipped with a 405 nm laser (Malvern UK), sCMOS camera and NTA software version 2.3, Build 0033 (Malvern UK)]. Before sample analysis, instrument performance was checked with silica 100 nm microspheres (Polysciences, Inc.). The 10K and 150K STB-EV pellets were individually diluted in fPBS to a range of 1/100,000. The samples were automatically injected into the sample chamber with a 1 ml syringe with the following script used for EV measurements: prime, delay 5, capture 60, repeat 4. Images of the analyzed samples were captured on camera at level 12 (Camera shutter speed; 15 milliseconds and Camera gain; 350) and NTA post-acquisition settings were optimized and kept constant between samples. Each video recording was analyzed to infer STB-EVs size and concentration profile.

***Western Blot Analysis***

We performed western blots on placental lysates (PL) and STB-EVs to further characterise and immune-phenotype. All STB-EVs pellets were probed with PLAP (for syncytiotrophoblast origin), CD63 and ALIX (to confirm the presence of extracellular vesicles), and Cytochrome C (as a negative EV marker) as recommended by the international society for extracellular vesicles (ISEV) (6). Following characterization and identification of extracellular vesicles in 10K and 150K STB-EV pellets, we renamed them to medium/large (m/l) and small (s) STB-EVs respectively.

**Table S1.** Table showing the antibodies used for Western blot.

| Antibodies | Concentration | Dilution | Antigen | Specificity | Manufacturer |
| --- | --- | --- | --- | --- | --- |
| Anti-PLAP (NDOG 2) | 1.6 µg/µl | 1/1000 | PLAP | STB-EV | In house antibody |
| Anti-CD63 | 200 µg/µl | 1/1000 | CD63 | STB-EV | Santa Cruz Biotechnology |
| Anti-ALIX | 200 µg/µl | 1/1000 | ALIX | S STB-EV | Cell Signalling |
| Anti-Cytochrome C | 200 µg/µl | 1/500 | Cytochrome C | Placenta homogenate | Santa Cruz Biotechnology |
| Polyclonal goat-anti-mouse/rabbit immunoglobulin HRP | | 1/2000 | Mouse and Rabbit Immunoglobulins | N/A | Dako UK Ltd |

***RNA-Sequencing Library Preparation and Sequencing***

For placental biopsy, 30 milligrams of placenta tissue were mechanically homogenized, and the RNA extracted with the RNeasy kit. An equal amount of STB-EV (200 µg) was diluted up to 200 µl. Total RNA was then extracted from this STB-EV mixture with the miRCURY™ RNA isolation kit. The extracted RNA was assessed for purity with Nanodrop (NanoDrop 1000 Software). After confirming that all samples had a 260/280 ratio greater than 1.8, the samples were sent to the Wellcome Centre for Human Genetics (WCHG) for sequencing. At WCHG, the RNA was assessed with a Bioanalyzer (Agilent Technologies) to determine the concentration and RIN values (for PL) which were all greater than 8 except the STB-EV samples with RIN between 1-3. It is widely accepted that the RIN values are not applicable for extracellular vesicles due to 1) the fragmented nature of RNA they contain and the limited presence of 18S and 28S ribosomal RNA peaks.

RNA libraries were prepared using the TruSeq-stranded total RNA (20020597) library preparation kits (Illumina) to generate reverse stranded paired reads. A standard procedure was followed for mRNA sequencing. Briefly, polyadenylated mRNAs were selected from total RNA samples using oligo-dT-conjugated magnetic beads. Poly-adenylated RNA samples were immediately converted into stranded Illumina sequencing libraries using 200 bp fragmentation and sequential adapter following the manufacturer’s specifications. The resulting cDNA was amplified, enriched, and indexed using 12 cycles of amplification with PCR primers, including an index sequence to allow for multiplexing. All RNA sequences were purified on gels and sequenced on a HiSeq2500 high output v3 flow cell using paired-end, 75 bp reads (Illumina).

***Taqman Gene expression assays***

Table S2. Table showing qPCR Primers, assay ID and amplicon length.

| **Gene Symbol** | **Assay ID** | **Amplicon Length** |
| --- | --- | --- |
| PAPP-A2 | Hs00535718_m1 | 67 |
| INHBA | Hs01081598_m1 | 61 |
| SIGLEC6 | Hs00971272_g1 | 117 |
| HTRA4 | Hs00538137_m1 | 75 |
| EBI3 | Hs01057148_m1 | 64 |
| FSTL3 | Hs00610505_m1 | 84 |
| HSD17B1 | Hs00166219_g1 | 124 |
| CGB3 | Hs00361224_gh | 55 |
| YWHAZ | Hs01122445_g1 | 62 |
| LEP | Hs00174877_m1 | 74 |
| COL17A1 | Hs00990036_m1 | 57 |
| FLNB | Hs00963202_m1 | 100 |
| SLC45A4 | Hs01088148_m1 | 70 |

***Supplementary results***

***Table S3. General characteristics of the STB-EV qPCR validation study population***

| Characteristics |  | Normal Pregnancy | Preeclampsia | P Value |
| --- | --- | --- | --- | --- |
| Sample size |  | 6 | 6 |  |
| Maternal age years (mean (SD)) |  | 35.50 (4.80) | 37.25 (4.11) | 0.600 |
| Body mass index kg/m^-2^ (mean (SD)) |  | 24.25 (3.30) | 34.88 (12.28) | 0.146 |
| **Systolic blood pressure mmHg (mean (SD))** |  | **109.50 (6.86)** | **183.75 (12.87)** | **<0.001** |
| **Diastolic blood pressure mmHg (mean (SD))** |  | **76.00 (1.41)** | **113.25 (9.71)** | **<0.001** |
| **Proteinuria plus(es) (mean (SD))** |  | **0** | **3.00 (0.82)** | **<0.001** |
| **Gestational age at delivery in weeks (mean (SD))** |  | **39.50 (0.58)** | **33.50 (3.00)** | **0.008** |
| **Birth weight (grams) (mean (SD))** |  | **3627.50 (247.98)** | **1808.75 (488.54)** | **0.001** |
| **Intrauterine growth restriction (IUGR) = Yes (%)^-^** |  | **0 (0)** | **4 (100.00)** | **0.034** |
| Male new-born gender (%) |  | 2 (50.00) | 1 (25.00) | 1.000 |

**
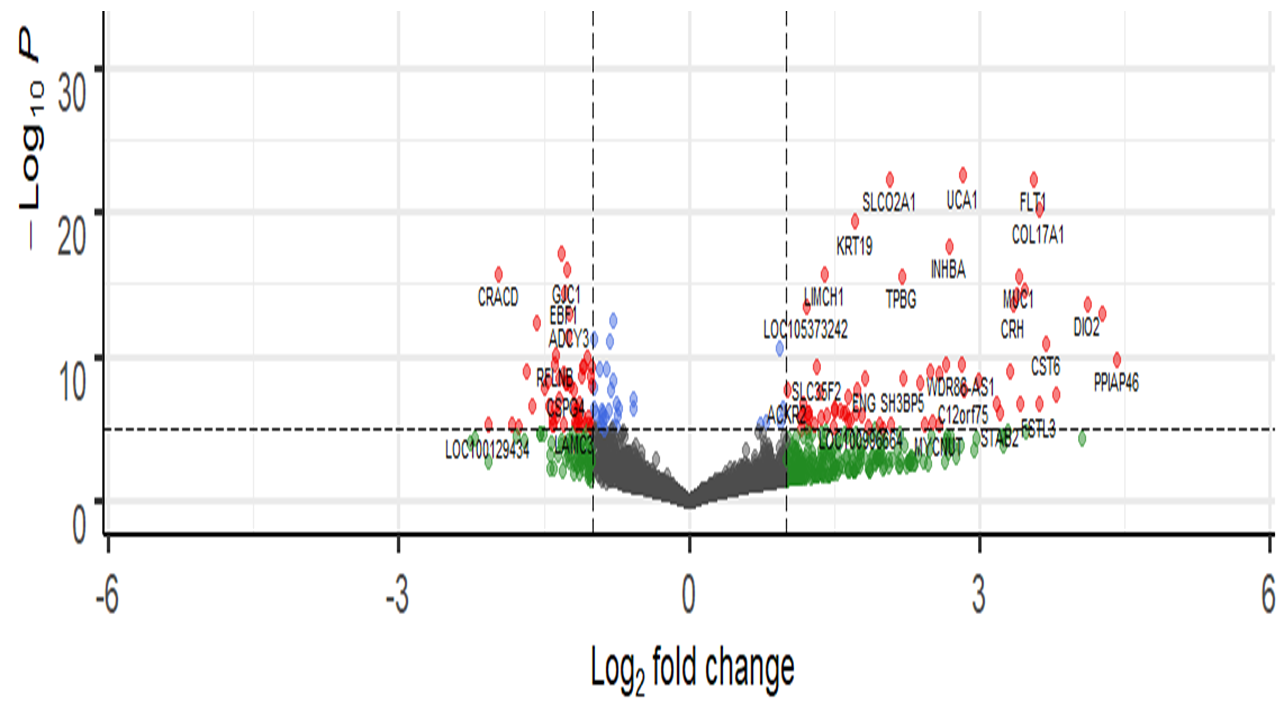
**

Figure S3. Volcano plot showing differentially expressed genes in the placenta. The most significantly upregulated genes are displayed in red on the right while the most significantly downregulated genes are displayed in red on the left.

**
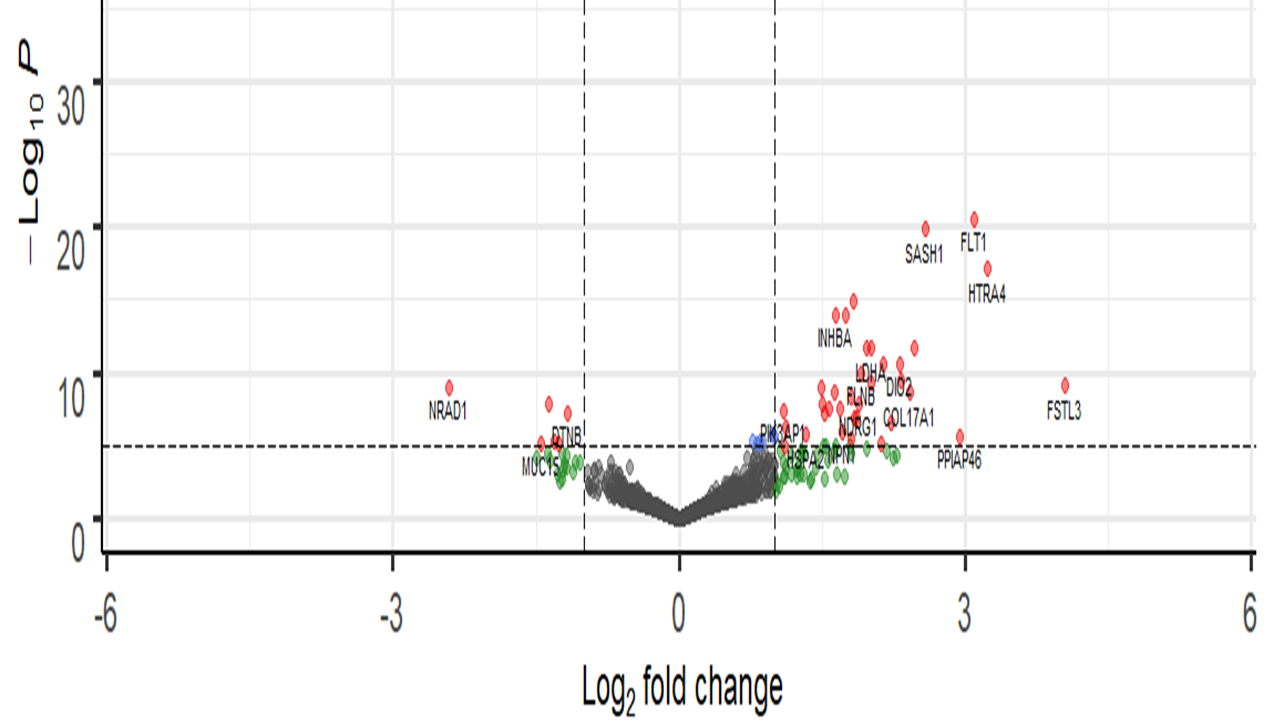
**

**Figure S4.** Volcano plot showing differentially carried genes in small STB-EVs. The most significantly upregulated genes are displayed in red on the right while the most significantly downregulated genes are displayed in red on the left.

**Table S4.** List of overlapping gene ontology (GO) terms and KEGG Pathways in the placenta, medium/large STB-EVs and small STB-EVs

| Sample Type | Gene Ontology terms and KEGG Pathway(s) |
| --- | --- |
| Biological Process (BP) | |
| Placenta and sSTB-EVs | Extracellular matrix organization |
|  | Extracellular structure organization |
|  | Cell-substrate adhesion |
|  | Regulation of vasculature development |
|  | Regulation of cell-substrate adhesion |
|  | Positive regulation of vasculature development |
|  | Cell-substrate junction assembly |
|  | Cell-matrix adhesion |
|  | Cell-substrate junction organization |
|  | Positive regulation of angiogenesis |
|  | Regulation of cell-matrix adhesion |
|  | Positive regulation of cell adhesion |
|  | Regulation of cellular response to growth factor stimulus |
|  | Negative regulation of locomotion |
|  | Endocrine process |
|  | Negative regulation of cell-matrix adhesion |
|  | Regulation of endocrine process |
|  | Transmembrane receptor protein serine/threonine kinase signalling pathway |
|  | Positive regulation of phosphatidylinositol 3-kinase signalling |
|  | Regulation of transmembrane receptor protein serine/threonine kinase signalling pathway |
|  | Endocrine hormone secretion |
|  | Response to transforming growth factor beta |
|  | Cellular response to transforming growth factor beta stimulus |
|  | Response to decreased oxygen levels, |
|  | Negative regulation of myeloid leukocyte differentiation |
|  | Regulation of phosphatidylinositol 3-kinase signalling |
|  | Response to hypoxia |
|  | Response to oxygen levels |
|  | Maintenance of location |
|  | Regulation of gonadotropin secretion |
|  | NAD metabolic process |
|  | Lipid localization |
| Placenta and m/lSTB-EVs | Platelet degranulation |
| Molecular Function (MF) | |
| Placenta and sSTB-EVs | Cell adhesion molecule binding, |
|  | Integrin binding |
| Cellular Component (CC) | |
| Placenta and sSTB-EVs | Collagen-containing extracellular matrix |
|  | Leading-edge membrane |
|  | Ruffle membrane |
|  | Cell-cell junction |
| m/lSTB-EVs and sSTB-EVs | Vesicle lumen |
| Placenta, m/l STB-EVs and sSTB-EVs | Actin cytoskeleton |
|  | Cytoplasmic vesicle lumen |
|  | Secretory granule lumen |
|  | Focal adhesion |
|  | Cell-substrate junction |
|  | Contractile fibre |
| Placenta and m/lSTB-EVs | Platelet alpha granule lumen |
| KEGG Pathways | |
| Placenta and sSTB-EVs | ECM-receptor interaction |
|  | Inflammatory mediator regulation of TRP channels |
|  | HIF-1 signalling pathway |
|  | Glycolysis/gluconeogenesis |
|  | Central carbon metabolism in cancer |
|  | Proteoglycans in cancer |
| Placenta, m/lSTB-EVs and sSTB-EVs | Focal adhesion |

**Major Resources Table**

**Antibodies**

| **Target antigen** | **Vendor or Source** | **Catalog #** | **Working concentration** | **Dilution** | **Persistent ID / URL** |
| --- | --- | --- | --- | --- | --- |
| PLAP | In house antibody |  | 1.6 µg/µl | 1/1000 |  |
| CD63 | Santa Cruz Biotechnology | sc-365604  RRID: AB_10847220 | 200 µg/µl | 1/1000 |  |
| ALIX | Cell Signaling | #2171  RRID: AB_1947944 | 200 µg/µl | 1/1000 |  |
| Cytochrome C | Santa Cruz Biotechnology | sc-13560  RRID: AB_627383 | 200 µg/µl | 1/500 |  |
| Mouse Immunoglobulins | Dako UK Ltd | P044701  RRID: AB_2617137 |  | 1/2000 |  |
| Rabbit Immunoglobulins | Dako UK Ltd | P044801  RRID: AB_2617138 |  | 1/2000 |  |

**Reverse transcription resources**

| **Target antigen** | **Vendor or Source** | **Catalog #** |
| --- | --- | --- |
| High-Capacity cDNA Reverse Transcription Kit with RNase Inhibitor | Applied Biosystems | 4374967 |
| TaqMan™ Fast Advanced Master Mix | Applied Biosystems | 4444558 |

**Flow cytometry resources**

| **Markers** | **Fluorochromes** | **Clone** | **CAT** | **Isotype** | **Dilution**  **/ Concentration** |
| --- | --- | --- | --- | --- | --- |
| CD41 | PE Vio770 | REA386 | 130-105-562  RRID: AB_2658049 | REA | 1 in 50 for dump channel analysis. |
| CD235 | PE Vio770 | RAE-175 | 130-100-258  RRID: AB_2656504 | REA |  |
| HLA Class-I (ABC) | PE Vio770 | REA230 | 130-101-460  RRID: AB_2652090 | REA |  |
| HLA Class-II (DRDPDQ) | PE Vio770 | RAE-332 | 130-104-828  RRID: AB_2652182 | REA |  |
| REA control |  |  |  | REA |  |
| CD41 | Pacific blue | HIP8 | 303713  RRID: AB_10681721 | Mouse IgG1 | 1 in 100 |
| CD235 | Pacific blue | HI264 | 349107  RRID: AB_11219199 | Mouse IgG2a | 1 in 100 |
| HLA Class-I (ABC) | Pacific blue | W6/32 | 311417  RRID: AB_493668 | Mouse IgG2a | 1 in 100 |
| PLAP | PE | Mouse mAb | N/A | IgG1 | 0.2μg/ml |
| IgG2a isotype control | Pacific blue | MOPC-173 | 981904 | IgG2a | 1 in 500 |
| IgG1 isotype control | Pacific blue | MOPC-21 | 400131  RRID: AB_2923473 | IgG1 | 1 in 200 |
| bioM | FITC | NA | NA | NA | 0.5-1nM |
